# Early detection of resistance to dual antiplatelet therapy in patients who have undergone percutaneous coronary intervention using the VerifyNow test and associated factors

**DOI:** 10.3892/mi.2024.180

**Published:** 2024-07-18

**Authors:** Song Giang Tran, Thi Kieu My Tran, Tan Sang Nguyen, Minh Phuong Vu

**Affiliations:** 1 Vietnam National Heart Institute, Bach Mai Hospital, Hanoi 11519, Vietnam; 2 Department of Hematology, Hanoi Medical University, Hanoi 11521, Vietnam; 3 Department of Coagulation, National Institute of Hematology and Blood Transfusion, Hanoi 11312, Vietnam; 4 Hematology and Blood Transfusion Center, Bach Mai Hospital, Hanoi 11519, Vietnam

**Keywords:** dual antiplatelet therapy, aspirin resistance, clopidogrel resistance, acute coronary syndromes, percutaneous coronary intervention, VerifyNow

## Abstract

Resistance to dual antiplatelet therapy (DAPT), including aspirin and clopidogrel, in patients who have undergone percutaneous coronary intervention (PCI) leads to the inability to prevent thrombotic complications. The present study aimed to evaluate early resistance to aspirin and clopidogrel in patients following PCI using the VerifyNow test and associated factors. A total of 50 patients diagnosed with acute coronary syndromes (ACS) who underwent emergency PCI and received DAPT were recruited in the present study. The detection of resistance to aspirin and clopidogrel was performed using the VerifyNow system. Resistance to aspirin was determined with VerifyNow Aspirin >550 aspirin reaction units (ARU). Resistance to clopidogrel was determined with VerifyNow P2Y12 >208 P2Y12 reaction units (PRU). The resistance rate to aspirin was 14%, while the resistance rate to clopidogrel was higher, at 34%. There were 2 patients with resistance to aspirin and clopidogrel (4%). Univariable logistic regression analysis revealed that diabetes, the use of β-blockers, and low levels of hemoglobin and hematocrit were associated with resistance to clopidogrel. Following multivariable logistic regression analysis, only the use of β-blockers was truly associated with resistance to clopidogrel. On the whole, the results of the present study may also prove to be helpful in the selection of therapeutic drugs for patients undergoing PCI and who are diagnosed with ACS.

## Introduction

Percutaneous coronary intervention (PCI) with drug-eluting stents (DES) in patients diagnosed with acute coronary syndromes (ACS) reduces restenosis compared with the use of bare-metal stents. However, due to its ability to delay endothelialization and increase platelet aggregation, the use of DES may be associated with an increased risk of developing stent thrombosis. Therefore, dual antiplatelet therapy (DAPT) is required to prevent thrombotic complications, thus reducing the risk of recurrent myocardial infarction (MI) or ischemic events. DAPT typically combines aspirin and one P2Y12 receptor inhibitor (clopidogrel, ticagrelor or prasugrel) (1,2).

To date, the optimal duration of DAPT following PCI remains controversial. Short-term DAPT following PCI (3-6 months) reduces the risk of bleeding. By contrast, long-term DAPT following PCI (≥12 months) reduces the risk of developing thrombosis and recurrent MI. Furthermore, long-term DAPT is recommended for patients without a high risk of bleeding (3-6). However, resistance to antiplatelet drugs (also known as high residual platelet reactivity) leads to the inability to prevent thrombotic complications, regardless of the duration of DAPT (7-9). Resistance to antiplatelet drugs is influenced by several factors, such as comorbidities, medications, genetic polymorphisms, and even race and ethnicity (10-15). Additionally, the concept of antiplatelet resistance includes clinical and laboratory resistance. Clinical antiplatelet resistance is defined as thrombotic complications that occur while patients are still taking regular antiplatelet drugs. Laboratory antiplatelet resistance is defined as the expected inhibition of platelet aggregation that is not detected by *in vitro* tests (16). Therefore, the determination of laboratory antiplatelet resistance may depend on the technique, sampling time and threshold (17-19). A previous study even demonstrated that this laboratory resistance to clopidogrel was 31% on day 5 following PCI and decreased to 15% on day 30 following PCI (18). Therefore, some authors prefer to use the term ‘high residual platelet reactivity’ or ‘high on-treatment platelet reactivity’ rather than ‘laboratory antiplatelet resistance’. The VerifyNow Assay is used to monitor the antiplatelet function of both aspirin and clopidogrel. This system measures platelet aggregation with fibrinogen coat beads in response to stimulants (acid arachidonic for aspirin and ADP for clopidogrel). Although the commonly accepted cut-off value for aspirin resistance is 550 aspirin reaction units (ARU), the cut-off value for clopidogrel, depending on the studies, ranges from 190-240 P2Y12 reaction units (PRU) or platelet inhibition ranges from 15-40% (2,19-23). This wide range of values may lead to differences in predicting thrombosis, as well as bleeding.

Due to the aforementioned complex influencing factors, although there are available studies (10-15) on resistance to DAPT following PCI, to date, this resistance remains a matter of concern. New reports of resistance to DAPT, particularly in other races, ethnicities and localities, are necessary for the development of appropriate treatment strategies for patients following PCI.

The present study aimed to evaluate the early laboratory resistance to aspirin and clopidogrel following PCI using the VerifyNow test and associated factors in Vietnamese patients.

## Patients and methods

*Patients*. The present retrospective cross-sectional study was conducted at the Vietnam National Heart Institute-Bach Mai Hospital and the National Institute of Hematology and Blood Transfusion, Hanoi, Vietnam, from October, 2020 to June, 2021. Only patients diagnosed with ACS who had undergone emergency percutaneous coronary intervention and the VerifyNow test for antiplatelet drug resistance were recruited in the present study. Patients with previous PCI, long-term antiplatelet therapy, a history of myeloproliferative syndromes, a platelet count <100x10^9^/l or >450x10^9^/l, a hematocrit level <29 l/l or >56 l/l, or a hemoglobin level <80 g/l were excluded from the study (19,20). The study protocol was approved by the Institutional Review Board of Hanoi Medical University (approval no. 5098/QĐ-ĐHYHN). The present study was a retrospective study, thus all data were obtained from medical records. Patient informed consent to participate was verbal All details of the patients were deidentified.

### Procedures

The patients underwent emergency PCI with DES and received DAPT, including aspirin and clopidogrel. The loading dose prior to PCI consisted of the oral administration of 325 mg aspirin and 600 mg clopidogrel, followed by a maintenance dose of 81 mg aspirin and 75 mg clopidogrel daily.

### VerifyNow test

The detection of resistance to aspirin and clopidogrel was performed using the VerifyNow system (Accriva Diagnostics, Inc.). Blood samples were collected 2-12 h after antiplatelet drug administration within 7 days following the PCI procedure. Resistance to aspirin (or a high residual platelet reactivity on aspirin) was determined with VerifyNow Aspirin >550 ARU. Resistance to clopidogrel (or a high residual platelet reactivity on clopidogrel) was determined with VerifyNow P2Y12 >208 PRU (19).

### Collected data

Blood cell indices, including hemoglobin, hematocrit, white blood cell count, platelet count and biochemical indices that were performed prior to PCI, were collected. Data on comorbidities, including diabetes, dyslipidemia, hypertension and previous stroke (cerebral vascular accident), status including smoking and overweight, medications including β-blockers, calcium channel blockers, angiotensin receptor blockers, angiotensin-converting enzyme inhibitors, statins and diuretics, were also collected.

### Statistical analysis

Data on the resistance to aspirin and clopidogrel are expressed as frequency and percentage. Qualitative variables such as comorbidities, status and medications, and quantitative variables, such as blood cell indices were compared between two groups according to the presence of antiplatelet drug resistance. The differences in qualitative variables were analyzed using the χ^2^ test or Fisher's exact test. Differences in quantitative variables were analyzed using the independent sample t-test (unpaired t test) or the Mann-Whitney U test according to their normal or non-normal distribution. Regression analysis using binary logistic was performed to determine the factors associated with the presence of resistance to the antiplatelet drug. SPSS 25 software (IBM Corp.) was used in statistical analysis. A value of P<0.05 was considered to indicate a statistically significant difference.

## Results

### Patient characteristics

A total of 50 patients were enrolled in the present study, including 14 females (28%) and 36 males (72%). The median age of the patients was 69 years (range, 45-91 years). The characteristics of the patients, including diagnosis, comorbidities, status, medications and laboratory indices are presented in Tables I and II. Patients diagnosed with MI [including non-ST elevation myocardial infarction (NSTEMI) (20%) and ST elevation myocardial infarction (STEMI) (34%)] accounted for the highest rate (54%). In particular, there were 5 patients with combined triple vessel coronary artery disease, accounting for 10% of the study population. There was also a high rate of patients with hypertension and dyslipidemia, and almost all patients used statins (Table I).

### Resistance to antiplatelet drugs and associated factors

The resistance rate to aspirin was 14%, while the resistance rate to clopidogrel was higher, at 34%, There were 2 patients with resistance to aspirin and clopidogrel (4%) (Table III).

Patients with resistance to clopidogrel had lower hemoglobin and hematocrit levels compared with patients without this resistance (P=0.004, 0.002; respectively; Table IV). The rate of resistance to clopidogrel in the group of patients using β-blockers was higher than that in the group not using β-blockers, with a statistically significant difference [9/16 (56.3%) vs. 8/34 (23.5%), P=0.023] (Table V). Logistic regression analysis using a single predictor (univariable analysis) was performed to determine the factors associated with resistance to clopidogrel. The factors were assessed for their association with resistance to clopidogrel, including hemoglobin, hematocrit, β-blocker use, and other factors, such as comorbidities (diabetes, dyslipidemia, hypertension and previous stroke) and medications (calcium channel blockers, angiotensin receptor blockers, angiotensin-converting enzyme inhibitors, statins and diuretics). This analysis revealed that four factors, such as diabetes, use of β-blockers, hemoglobin and hematocrit, were associated with resistance to clopidogrel (Table VI). Among the hemoglobin and hematocrit variables, B coefficients were <0; thus, low hemoglobin and hematocrit were associated with resistance to clopidogrel. Logistic regression analysis using multiple predictors (multivariable analysis) was performed to adjust for independent variables in the model and it was determined that only the use of β-blockers use was associated with the presence of resistance to clopidogrel (Table VII). Adjusting for sex and age in the logistic regression model also revealed that the use of β-blockers was truly an associated factor (Table VIII).

## Discussion

The prevalence of antiplatelet drug resistance varied according to various studies. In patients with cardiovascular disease, the rate of laboratory resistance to aspirin ranged from 2-57%, while the range of resistance to clopidogrel was 16-50% (24,25). Resistance to an antiplatelet drug is influenced by several factors including race, ethnicity and geographic region. Ebrahimi *et al* (26) revealed that there was a significant increase in aspirin resistance rates according to the geographic background and the prevalence in Asia and Europe was reported to be higher than in Africa and America (27.3 and 25.7% vs. 19.5 and 19.1%). Infeld *et al* (27) demonstrated that in European Americans, platelet reactivity on aspirin treatment was higher than that in African Americans. On the other hand, Pendyala *et al* (13) determined that African Americans have a higher prevalence of high platelet reactivity on clopidogrel treatment. The polymorphism of the platelet gene is associated with residual platelet reactivity on treatment; thus, race may affect the resistance to antiplatelet drugs. Genetic polymorphisms that contribute to resistance to aspirin include *COX-1* polymorphisms of C50 T, -A842G and A1676G; the *COX-2* polymorphism of -765, while genetic polymorphisms associated with clopidogrel resistance were CYP2C19*2, CYP2C19*3 and CYP2C19*17 (2,12). In the present study, Vietnamese patients had an aspirin resistance rate of 14%, which was lower than that reported in the study by Ebrahimi *et al* (26) in the Asian patient group, but greater than that in the study by Chadha *et al* (28) in the Indian patient group with an aspirin resistance rate of 2%. On the other hand, Akkaif *et al* (14) and Hasan *et al* (29) reported that, due to the genetic polymorphism with a high prevalence of CYP2C19*2, CYP2C19*3 and CYP2C19*17, Asians are at a higher risk of developing resistance to clopidogrel. The present study also demonstrated that the resistance rate to clopidogrel was moderately high, at 34%. This is critical for developing appropriate treatment strategies for Asian patients. However, the sample size in the present study was small. Further research is required to determine these resistance rates.

In addition to genetic polymorphisms, other factors were considered to be associated with resistance to antiplatelet drugs. β-blockers are often prescribed to patients with coronary artery disease due to their beneficial features, such as reduced myocardial oxygen demand, inhibited arrhythmias and relieved pain (30). However, the influence of β-blockers on resistance to clopidogrel remains controversial. Bonten *et al* (31) demonstrated that β-blockers decreased platelet aggregation. On the contrary, Zhang *et al* (32) considered that β-blockers reduced the risk of bleeding in patients with ACS or undergoing PCI. Su *et al* (33) demonstrated that ß-blockers were associated with resistance to clopidogrel. Similar to the study by Su *et al* (33), the present study also found that the use of β-blockers was associated with clopidogrel resistance. Although the sample size in the present study was small, this observation may also be helpful in the selection of treatment drugs for patients after PCI.

In the present study, univariable logistic regression analysis revealed that the clopidogrel resistance rate was associated with diabetes, low hemoglobin and hematocrit. Diabetes was considered a factor associated with resistance to clopidogrel. Hyperglycemia, the deficiency of intrinsic insulin or insulin resistance increases platelet reactivity due to the loss of inhibition of P2Y12 by the insulin-related pathway. However, Niijima *et al* (34) and Sweeny *et al* (35) demonstrated that prasugrel or ticagrelor were more effective than clopidogrel in patients with diabetes. However, in the present study, the multivariable logistic regression analysis did not determine that diabetes was a truly independent factor associated with resistance to clopidogrel.

Kakouros *et al* (36) and Kim *et al* (37) demonstrated that the hematocrit altered the VerifyNow P2Y12 results. Kim *et al* (38) also claimed that the association between hemoglobin and high residual platelet reactivity on clopidogrel may be due to laboratory errors. However, Janssen *et al* (39) suggested that even adjusting the PRU cut-off value according to hematocrit did not improve the ability to predict thrombotic events after PCI. VerifyNow has the advantage of using whole blood and a small sample volume; however, this advantage can lead to the aforementioned consequences. However, in the present study, following logistic regression analysis using multiple predictors, hematocrit and hemoglobin were not shown to be associated with clopidogrel resistance.

In addition, the cut-off value for resistance to clopidogrel ranges from 190-240 PRU. The present study used the cut-off value of 208 PRU according to Stone *et al* (19). Using a low cut-off value may lead to unnecessary adjustments in antiplatelet therapy. Using a high cut-off value can delay the detection of thrombotic risk. Patients with laboratory antiplatelet resistance, which is detected by VerifyNow tests, should be monitored and repeated tests. If possible, genetic polymorphisms should be performed to draw accurate conclusions.

In general, further evidence is required to confirm that a factor (apart from genetic polymorphisms) is associated with resistance to clopidogrel. However, in the present study, the authors did not find any factors associated with aspirin resistance.

The present study had several limitations, including a sample size and the cross-sectional study design. Moreover, the patients were not monitored to detect thrombotic events and VerifyNow was not repeated periodically.

In conclusion, the present study demonstrated that the resistance rate to clopidogrel was higher than the resistance rate to aspirin in Vietnamese patients with ACS who underwent percutanous coronary intervention. The use of β-blockers may be associated with resistance to clopidogrel. However, further research is required to determine factors that are associated with resistance to antiplatelet drugs, including aspirin and clopidogrel, to develop an appropriate treatment strategy. In the future, the authors hope to conduct studies with large sample sizes and long follow-up periods to be able to design a process to closely monitor antiplatelet drug resistance to limit thrombotic events.

## Figures and Tables

**Table I:** Characteristics of the patients in the present study.

Characteristics	No. of patients	Percentage
Classification of CAD		
Unstable angina	18	36.0
MI		
NSTEMI	10	20.0
STEMI	17	34.0
Triple-vessel CAD	5	10.0
Comorbidities and status		
Hypertension	34	68.0
Dyslipidemia	36	72.0
Diabetes	12	24.0
Previous stroke (CVA)	3	6.0
Overweight	23	46.0
Smoking	19	38.0
Drugs		
β-blocker	16	32.0
Calcium channel blocker	6	12.0
ACE inhibitors, ARBs	33	66.0
Statins	49	98.0
Diuretics	14	28.0

ACS, acute coronary syndrome; MI, myocardial infarction; NSTEMI, non ST elevation myocardial infarction; STEMI, ST elevation myocardial infarction; CAD, coronary artery disease; CVA, cerebrovascular accident; ACE, angiotensin-converting enzyme; ARBs, angiotensin II receptor blockers.

**Table II:** Values of laboratory indices.

Index	Mean	SD
Hemoglobin (g/l)	134.98	15.04
Hematocrit (l/l)	40.33	4.18
WBC count (x10^9^/l)	9.36	3.29
Platelet count (x10^9^/l)	260.72	70.08
ARU	456.50	71.23
PRU	164.94	93.66
Troponin-Ths (ng/l)	1832.81	4918.36
Glucose (mmol/l)	7.56	3.26
Creatinine (µmol/l)	106.04	102.39
AST (U/l)	74.66	96.68
ALT (U/l)	31.97	29.08

ARU, aspirin reaction unit; PRU, P2Y12 reaction unit; AST, aspartate transaminase; ALT, alanine transaminase.

**Table III:** Presence of aspirin and clopidogrel resistance.

Characteristic	No. of patients	Percentage
Aspirin resistance	7	14.0
Clopidogrel resistance	17	34.0
Resistance to 1 drug	20	40.0
Resistance to 2 drugs	2	4.0

**Table IV:** Red blood cell indices according to the presence of antiplatelet resistance.

	Aspirin resistance		Clopidogrel resistance		
Index	No (n=43)	Yes (n=7)	No (n=33)	Yes (n=17)	P-value
Hemoglobin (g/l)	134.30±14.67	139.14±17.80	139.30±12.15	126.59±16.84	P1>0.05
(mean ± SD)					P2=0.004
Hematocrit (l/l)	40.27±4.22	40.73±4.19	41.58±3.43	37.91±4.53	P1>0.05
(mean ± SD)					P2=0.002

P1, between patients with aspirin resistance and those without aspirin resistance; P2, between patients with clopidogrel resistance and those without clopidogrel resistance.

**Table V:** Frequency of the presence of antiplatelet resistance in patients with or without β-blocker use.

	Aspirin resistance		Clopidogrel resistance		
Factor	No (n=43)	Yes (n=7)	No (n=33)	Yes (n=17)	P-value
β-blocker use					
No (n=34)	28	6	26	8	P1>0.05
Yes (n=16)	15	1	7	9	P2=0.023

P1, between patients with aspirin resistance and those without aspirin resistance; P2, between patients with clopidogrel resistance and those without clopidogrel resistance.

**Table VI:** Univariate logistic regression analysis for factors associated with the presence of clopidogrel resistance.

Factor	B	SE	Wald	P-value	OR	95% CI
Diabetes	1.430	0.646	4.899	0.048	3.920	1.010-15.210
β-blockers	1.430	0.646	4.899	0.027	4.179	1.178-14.824
Hemoglobin	-0.067	0.026	6.707	0.010	0.935	0.889-0.984
Hematocrit	-0.251	0.094	7.172	0.007	0.778	0.648-0.935

SE, standard error; OR, odds ratio; CI, confidence interval.

**Table VII:** Multivariate logistic regression analysis for factors associated with the presence of clopidogrel resistance.

Factor	B	SE	Wald	P-value	OR	95% CI
Diabetes	0.143	0.913	0.024	0.876	1.154	0.193-6.912
β-blockers	2.317	0.868	7.120	**0.008**	**10.149**	**1.850-55.675**
Hemoglobin	-0.011	0.098	0.012	0.912	0.989	0.816-1.199
Hematocrit	-0.288	0.370	0.605	0.437	0.750	0.363-1.549

Values in bold font indicate statistically significant differences. SE, standard error; OR, odds ratio; CI, confidence interval.

**Table VIII:** Association between the use of β-blockers and the presence of clopidogrel resistance with adjusted with sex and age.

Factor	B	SE	Wald	P-value	OR	95% CI
β-blockers	1.375	0.661	4.336	0.037	3.957	1.084-14.441

SE, standard error; OR, odds ratio; CI, confidence interval.

## Data Availability

The datasets used and/or analyzed during the current study are available from the corresponding author on reasonable request.
